# Food Waste in Primary Production: Milk Loss With Mitigation Potentials

**DOI:** 10.3389/fnut.2019.00173

**Published:** 2019-11-12

**Authors:** Margaret D. March, Luiza Toma, Bethan Thompson, Marie J. Haskell

**Affiliations:** Scotland's Rural College Research, Edinburgh, United Kingdom

**Keywords:** dairy, farm, milk loss, LCA, carbon footprint, antibiotics

## Abstract

Sources and quantities of milk loss in primary production are presented in this paper through an analysis of results from a 2018 survey. Responses from 43 dairy farms in Scotland showed that milk losses occurred due to withdrawal periods for veterinary treatment, parlor infrastructure, and lapses in management routine. A partial life cycle assessment detailed flows of milk from cow to farm gate and captured farm inputs such as imported feeds and fertilizers. Incidence of animal health events such as mastitis, that routinely lead to milk withdrawal were quantified alongside strategies carried out by farmers to reduce milk loss. Treatment for mastitis accounted for 76% of all milk withdrawal days and the remaining 24% stemmed from therapies for health events such as uterine disorders and lameness. Withdrawal periods for mastitis treatments averaged 4.5 days, with a mean incidence of 20% of cows in a herd. Across all farms, an average of 98.2% of total milk produced was sold, 0.66% was purposely retained, 0.55% was rejected due to antibiotic residues, 0.5% was lost from parlor to bulk tank infrastructure and a further 0.09% was rejected by the processor. Carbon footprints found greenhouse gas (GHG) emissions averaged 0.849 kg CO_2_e/kg across farms for the milking herd. A scenario of 20% fewer withdrawal days reduced GHG's on average by 0.6%. Additional mitigation was attained by reductions in milk loss from parlor infrastructure and the bulk tank, and this showed a 1% reduction in GHG emissions could be achieved through higher volumes of milk sales. Categorizing responses by management system type highlighted differences in proportional losses between all year round housed and conventional grazing regimes. The most predominant health disorder leading to milk withdrawal was mastitis, however losses due to other health events and parlor infrastructure were not insignificant on Scottish dairy farms.

## Introduction

While 821 million people on earth are hungry, 1.3 billion tons of edible food is estimated to be lost or wasted, which equates to one third of all food produced globally (1). These circumstances have propelled an interest in reducing losses and waste along the whole food supply chain (FSC), as a means to improving food security. Food waste in the European Union (EU) is estimated to total 88 million tons, or 173 kg per capita per year (2). Food and drink waste estimates for Scotland in 2013 totaled 1.35 million tons, or 252 kg per capita (3). Neither of these estimates include losses stemming from primary production, because of a lack of sufficient data (3). Studies that consider primary production are limited, and those that are available are considered outdated (1, 4–6). Evidence suggests that losses in primary production are not trivial, further knowledge is required and more specifically, primary production waste estimates for products at a national level be quantified (7).

One reason for a lack of primary production data is that food losses and waste at this stage of the supply chain have proven difficult to define. Until recently this has been an issue across food waste studies in general with a plethora of accounting methods and terminology used, which have impeded the comparability of studies (8). In the last decade however there has been a move to harmonize methodologies and standardize accounting practices (9). Guidelines to quantify food waste totals for EU nations, developed through the FUSIONS project, and the Food Loss and Waste Accounting and Reporting Standard (FLW), used to quantify and attribute food waste globally, adopt different approaches to scope (10). This has resulted in inventory differences and gaps in accounting for waste in primary production (6, 7). Consensus on definition and boundaries applied to studies focusing on primary production is yet to be achieved (7). A system boundary for primary production in dairy farming can be defined from when the milk is drawn from the cow to when the milk is delivered to the processor (7). However, milk that leaves the farm gate can still be rejected at the processor gate and sent for incineration if a tanker delivery fails for reasons such as residue limits being exceeded.

Available estimates of milk loss in the literature differ in scope. Estimates of milk production not actually attained by a cow because of illnesses such as mastitis, are included and added to actual physical losses. The FAO suggests milk not collected and sold due to cow illness and unproduced loss is 3.5%. Studies from Nordic countries which focus on actual milk loss due to disease apply the FUSIONs 0.3% as an estimate of milk waste (11, 12). Small samples of Swedish and Finnish farmers estimated they discarded 0.32 and 0.5% of milk produced, respectively, due to antibiotic residues (13, 14). In France, milk loss stemming from farm production to processing was estimated to range from 5.6 to 8.2%, with 3.2% attributed to primary production losses (4, 15).

Dairy cows receive antibiotic treatments for health disorders such as mastitis and metritis (16). Treatments have consequences for food loss and waste but also for environmental outcomes (17, 18). In the EU the European Medicines Agency (EMA) determines maximum residue limits in milk and farmers required to withdraw milk from sale routinely discard it on the premises. On some farms, and generally for financial or convenience reasons, rejected milk is fed to pre-weaned calves (16, 19). This practice has been shown to increase fecal shedding of antimicrobial resistant bacteria (20, 21) and one study found gut bacteria of calves to have increased resistance after consuming milk containing penicillin (22). In the EU, antimicrobials consumed by food producing animals accounts for 70% of the usage of these substances, more than double the amount for humans (23). Reductions in antibiotic use of 12 and 22% were achieved in the EU and UK, respectively, from 2011 to 2014 (24), and one current reduction target for the UK dairy sector is a 20% decrease in total usage (25).

In terms of environmental outcomes, food production is estimated to be attributed to 10–12% of GHG emissions globally (26) and UK and western EU emissions stemming from milk production are 1.2 and 1.4 kg CO_2_ e/kg FPCM, respectively (27, 28). These figures are lower than the global average of 2.5 kg CO_2_ e/kg FPCM, nevertheless, measures to decrease milk waste will reduce CO_2_ emissions. Decreasing losses in primary production should also exert a positive influence on food security.

This study has two objectives, firstly to provide an assessment of milk loss in primary production on dairy farms in Scotland and secondly to estimate possible reductions in GHG emissions corresponding with (a) a 20% reduction in the use of antibiotics; and (b) an additional 50% reduction in milk losses from infrastructure. Flows of milk, from cow to uplift, to farm and processor gate were gauged by survey responses with all possible losses stemming from health disorders and other causes included, whether the loss was intended or not. Detailed information allowed a comprehensive assessment and identification of possible hotspots where reductions in milk loss could be achieved. A 20% reduction was applied as it is equivalent to current targets for the UK dairy sector (25).

## Materials and Methods

### Data Collection and Analysis

A scoping study was carried out using historical Langhill data (29) combined with investigations on SRUC dairy farms to identify potential areas of milk loss. A survey was designed to enable a partial life cycle assessment (LCA) of milk loss stemming from multiple sources on farm. Survey questions aimed to determine the influence of factors such as incidence and type of disease on amounts of milk rejected by farmers. Data relating to potential sources of farm milk loss, production characteristics, feed and fertilizer use, labor, animal health and behavior were collected predominantly via face to face interviews. A pilot survey was carried out at SRUC's Crichton Farm prior to contacting farmers to assess the ease at which responses could be given. Survey questions are provided for reference as supplementary material.

Social Research Approval was gained from Scottish Government (SG) who supplied contact details of 150 dairy farms across Scotland with a herd size >25 milking cows. Letters were sent to 150 farmers to provide an opportunity to opt out of the process. Farmers who didn't opt out were contacted by telephone. A total of 56 possible respondents opted out giving reasons such as they did not have time, however 10 farms were no longer milking cows. Respondents not included in the SG list were contacted through farmers already participating in the survey and a total of 43 interviews were carried out.

A dataset of the 43 survey responses was compiled and analyzed in Excel to create an inventory of annual dairy farm inputs and outputs, with a focus on quantities and reasons for milk loss. Inventory data included respondent attributes such as gender, age, education, succession plans and farm characteristics, for example herd size, average annual yield, calving pattern, replacement rates, and milking regime. Management systems were described as AYR housed, part summer housed or composite. Cows managed in a composite system are housed in the winter and grazed in the summer. Incidence of mastitis, uterine and other health issues such as lameness with accompanying withdrawal periods per cow were recorded to capture all possible disease types whose treatment can lead to milk withdrawal.

Outputs of milk sold, average annual yield per cow, and fat and protein contents were recorded for each farm alongside hygiene indicators such as somatic cell count. Incidence of disease by type and the number of withdrawal days were provided by respondents. Quantities of milk rejected from sale due to milk withdrawal periods required for veterinary treatment, or milk not sold because of a high somatic cell count were reported for each farm. The destination of milk rejected from sale was provided and included quantities of milk fed to calves. Farmers indicated the types of actions they were taking, or could take, to reduce the incidence of health issues leading to milk withdrawal on farm. Respondents were then asked to rate the effectiveness of any actions implemented. Quantities of milk voluntarily retained for calf feeding or for human consumption on farm were recorded. The method of feeding colostrum was identified as directly, indirectly (from frozen), and sold.

Losses from infrastructure, spillages, accidents and bulk tank rejections with reasons and associated penalties were recorded. Respondents provided losses from the farm bulk tank that had occurred in the last year due to lapses in management routine. Rejection by a processor for reasons such as temperature and antibiotic residue were reported over a 5 year period. This was because bulk tanks rejected by a processor did not occur on all farms and were not generally an annual occurrence. Processor rejections were annualized using bulk tank capacity, corresponding to those respondents reporting losses. A bulk tank rejected due to the presence of antibiotic residues would ordinarily be detected upon delivery of a tanker to a processor.

### Inventory

**Figure 1:**
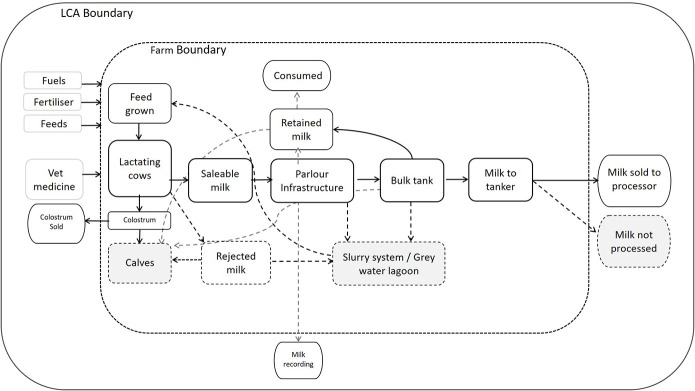
Boundaries considered, and flows of milk through a typical dairy farm.

LCA is used to determine environmental or other impacts along a product chain by compiling an inventory which encompasses system inputs. LCA is described by international standard ISO:14040 (30) and follows a specific methodical framework consisting of four phases from scope and boundary setting to inventory analysis and impact assessment. Furthermore, where a system delivers more than one product, an allocation methodology can be applied and attributed to each output (30). A “farm gate” boundary used in this study included inputs and outputs related to milk production on farm and avoided complications that can arise once milk leaves a farm (31). Figure 1 shows the LCA and farm boundaries alongside inputs, outputs and flows of milk through a typical dairy farm.

Functional units, within an LCA, can be defined as a quantifiable measure of the value of the studied system from which the input and output data can be normalized (30). The primary function of a dairy farm is milk production, thus, to account for milk quality and allow meaningful comparisons, fat and protein corrected milk yield (FPCM) is calculated using the following equation (32).

$$\begin{array}{l} \text{FPCM }\left(\text{kg}\right)=\text{Production }(\text{kg}/\text{year})\times [0.1226\times \text{Fat }(\%)\\ \;+0.0776\times \text{Protein }(\%)+\;0.2534] \end{array}$$

### Impact Assessment and Scenarios for Comparison

Results from the inventory were analyzed and scenarios were established to understand milk loss in terms of environmental impact by classifying and modeling the inputs and outputs for each category in terms of Global Warming Potential (GWP) (30). Emissions attributed to climate change are reported in CO_2_ equivalents per kg of FPCM output. Carbon footprints, for each of the surveyed dairy farms were estimated using SAC's AgRECalc v1.4 (33) a carbon foot-printing and resource efficiency tool which utilizes IPCC (34) methodology with a PAS2050 (35) accredited version available online. Tier II emission factors are applied for livestock and manure management and Tier I for fertilizer and crop residue N_2_O (36).

Farm footprint input variables comprised of milking cows, dry cows, purchased concentrate, forage crops grown, application of fertilizers, and diesel and electricity consumption. Farm purchased concentrates were assumed to be nutritionally similar, with an emission factor of 200 kg CO_2_e/ton and diet digestibility of 74% (37, 38). Fresh weight yields of silage, wheat and barley grown in Scotland were estimated to be 21, 12, and 11.6 t/ha, respectively (39). Electricity use was estimated using milk yield because a proportion of farms surveyed operated a mixed farm type (37). Percentages of time allocated to manure management at pasture, slurry or solid manure were estimated using dairy management system type, replacement rate and calving interval. Six surveys were excluded from the carbon foot-printing exercise, as some input variables were incomplete.

Baseline carbon footprints were calculated for each farm and two scenarios were carried out to estimate mitigation potentials. Scenario 1 corresponded to a 20% reduction in the use of antibiotics and was modeled by a corresponding reduction in withdrawal days being brought about by disease prevention. A 20% decrease in farm antibiotic use is equivalent to current targets for UK dairy farms (25). Scenario 2 added a 50% decrease in losses from parlor infrastructure and bulk tank losses brought about through technology and management practices. Production emissions related to specific types of veterinary pharmaceuticals, and their effects once they have been released to the environment, are not routinely included. The farm gate milk price applied to consider the financial effects of increased milk sales was 29.2 p/liter, which represented average farm gate milk price paid between April 2017 and March 2018 (40). Effects of management system on the incidence of disease and milk withdrawal were investigated.

## Results

The first section outlines survey responses and summarizes flows of milk produced on Scottish dairy farms. The second section examines the environmental and financial impacts of scenarios to reduce milk loss on farm.

### Survey Response and Analysis

Respondents were located across Scotland from Galloway in the southwest to Orkney in the north. The majority of completed surveys arose from areas of higher milk production, such as Dumfries and Galloway and Ayrshire. The forty three completed surveys represent approximately 5% of a total of 902 dairy farms remaining in Scotland (41). Descriptive statistics of selected farm characteristics are shown in Table 1. Most survey respondents were male (90%), with only 4 surveys being completed by female dairy farmers. Age groups ranged from under 35 years to over 65 years with over a third of respondents being aged between 45 and 54. Half of all respondents attended education to college level, 12% held university degrees, and 38% did not attend college or university. Three quarters of respondents were owners of their business, while 7% were tenants and 7% were managers. Almost two thirds of respondents inherited their business and half of all respondents had identified a successor.

**Table 1:** Selected general characteristics of farms participating in the survey.

	**Unit**	**Mean**	**Standard error**	**Minimum**	**Maximum**	***n***
Yield	liters	8,315	254	5,000	12,000	43
Fat	%	4.1	0.02	3.9	4.5	43
Protein	%	3.3	0.03	2.9	4.0	43
Lactating cows	n	176	15.5	42	520	43
Dry cows	n	29	2.6	10	80	42
Milking herd	n	205	17.7	56	580	43
Calving interval	days	402	2.9	370	450	38
Farmed land	ha	205	20.0	44	595	43
Grazing	ha	74	9.3	14	200	29
Cut and grazed	ha	93	9.7	25	283	36
Silage /milking cow	kg /day	32	1.6	10	50	41
Silage DM	%	27	0.5	20	35	40
Replacement rate	%	26	1.1	11	46	41
Bulk tank SCC	000's	160	12.5	12	581	43

*DM, Dry Matter; SCC, Somatic Cell Count*.

Holstein Friesian cows were kept by 60% of respondents, and Holstein and Ayrshire cows were the main breeds for 21 and 10% of dairy farmers, respectively. There was a mix of farm types in the sample, with over 30% of responses arising from specialist dairy farms and dairy/beef, dairy/sheep, dairy mixed, comprised 26, 17, and 23%, respectively. Management systems were categorized by farmers as composite (summer grazed winter housed), AYR housed and part summer/high yielder housed and grazing only representing, 49, 28, and 23%, respectively. Mean annual yield per cow averaged across all farms was 8,315 liters. The preferred calving pattern was all year round (AYR), accounting for 93% of respondents and the average calving interval was 402 days (Table 1). Some survey questions were not applicable to all respondents, for example, grazing land for cows being housed AYR. Not all respondents recorded measurements such as the dry matter (DM) of forage crops such as silage. No respondents operated with a grazing only management system, this was not surprising, as grass does not grow sufficiently AYR in Scotland.

### Milk Loss

Milk was withdrawn from sale due to treatments given to animals for a range of health events. Mastitis, uterine, and lameness and other disorders were attributed to 76, 8, and 16% of all withdrawal days, respectively. Mastitis was the most prevalent disease reported, with a 20% incidence. Withdrawal periods for mastitis were, on average, higher than for other diseases at 5.6 days (Table 2). Uterine, lameness and other health disorders requiring milk to be removed from sale accounted for nearly ¼ of total withdrawal days. Dry cow treatment and dry cow teat sealants were given to cows on 98% of farms. These treatments were provided selectively on 49% of farms. Average incidence of high somatic cell count (SCC) was 9% of cows. Twenty three percent of farmers surveyed reported excluding high SCC milk, for a period averaging 4.5 days.

**Table 2:** Disease incidence and associated withdrawal days average and standard deviation.

	**Incidence rate of disease**			**Milk withdrawal (days)**	
	**Mean**	**Standard deviation (SD)**	**Range**	**Mean**	**Standard deviation (SD)**
Mastitis	0.20	0.02	0.70	5.6	0.33
Uterine disorders	0.07	0.01	0.22	2.8	0.51
Lameness and other	0.07	0.01	0.31	4.3	0.30

Milk produced on farm may not be sold for a variety of reasons which can be beneficial or detrimental to the enterprise (Figure 1). Total milk production on each farm was calculated by adding the volume of milk sold to quantities of milk retained, milk rejected and milk lost due to infrastructure or other problems. Table 3 provides descriptive statistics showing average proportions of milk produced, sold, retained, and lost on the sampled dairy farms along with the destination of that milk. Milk voluntarily unsold is retained by the farmer, for human consumption or for calf feeding. Across all surveyed farms, the proportion of milk retained was, on average, 0.7% of total milk production (Table 3). However, over 80% of respondents purposely retained milk for consumption. Farmers consumed 1,027 liters per year on average and fed an average of 15,807 liters to calves, which represented 90% of all milk retained.

**Table 3:** Descriptive statistics of gross milk production, sales, and proportional loss on farm.

	**Mean**	**Standard error**	**Minimum**	**Maximum**	**Destination**
Gross production FPCM (kg)	1,729,892	187,701	233,034	5,882,571	
Sold (%)	98.2	0.002	0.929	0.997	Processor
Retained (%)	0.66	0.002	0	0.047	Consumed on farm
Infrastructure losses (%)	0.46	0.002	0	0.055	Calves / slurry system
Rejected by farmer (%)	0.55	0.001	0	0.022	Calves / slurry system
Bulk tank losses (%)	0.04	0.0002	0	0.007	Slurry system
Rejected by processor (%)	0.09	0.0002	0	0.004	Slurry/waste disposal

*FPCM, Fat and protein corrected milk*.

Quantities of milk rejected because of antibiotic residues arising from veterinary treatment of health disorders were reported by 93% of respondents. Three respondents provided no estimate of amounts of rejected milk even though they had provided disease incidences and withdrawal days for their herds. On average, rejected milk represented 0.6% of total production. Nineteen percent of respondents fed their rejected milk to calves. Total milk rejection on farm averaged 10,750 liters per holding per year, or 43 liters per cow per year and the majority of farmers disposed of rejected milk into the slurry system.

Infrastructure losses of at least one type were reported by 93% of respondents. Total infrastructure losses of all types averaged 3.5 tons per year per farm, or an average of 0.46% of all milk produced. Infrastructure losses were reported to stem from the filter (62%), buffer tank (22%), and from washing through pipes, which was generally carried out after each milking. Losses from spills and accidents were wide ranging. This loss type was reported as never or rarely happening on some farms to happening every 6–8 weeks to daily on others. Zero losses of this type were reported by two respondents, who described feeding all infrastructure and washed through milk to their calves. Respondents indicated that spillages and accidents were generally caused by lapses in management routine and the milk was destined for the slurry system.

Bulk tank losses occurred on 16% of farms and averaged 4,025 liters annually. Farmers indicated that bulk tank losses were caused by lapses in management, such as forgetting to connect pipework or by mistakenly allowing milk containing antibiotic residue to enter the bulk tank. Two farmers who suspected their bulk tank may fail due to antibiotics disposed of their milk in the slurry system to avoid a fine or penalty from the processor. Bulk tank losses were also reported to occur when transferring milk to the tanker, during power cuts, or in extreme winter weather if the tanker could not uplift.

Bulk tank rejections by a processor were reported by 53% of respondents. These rejections were caused by failures of equipment such as a compressor serving the cooling system, or by contamination due to antibiotic residue. Milk rejection because of raised temperature occurred in 35% of bulk tank loss cases and was disposed of into the slurry system before being uplifted. Rejection for antibiotic residue was reported in 65% of cases and usually incurred a penalty in addition to loss of income from milk sales. An annualized average of 2.0 tons of FPCM was estimated to be lost due to antibiotic residue. Penalties can be avoided if a farmer reports a failure to the processor prior to uplift. Frequency of bulk tank rejection ranged from once every 5 years to an annual occurrence. Bulk tank milk containing antibiotic residues would be detected at the processor gate prior to discharging milk from the tanker. A failed tanker would be treated as hazardous waste and disposed of by incineration at a specialist site.

Milk recording was noted by four respondents as a flow of milk from a farm. One respondent reporting a loss of 720 liters/year. Milk recording is carried out on 73% of herds across Scotland, on ~131,331 cows and each recording requires 35 ml per cow (pers. comm. J. Mathie, Cattle Information Service). Milk recording can be viewed as a voluntary milk retention that aids farm management and herd health. Colostrum was fed directly to the calf by 65% of farmers and was reported to be sold by 1/3 of respondents, for processing into powder.

### Impact Assessment

**Figure 2:**
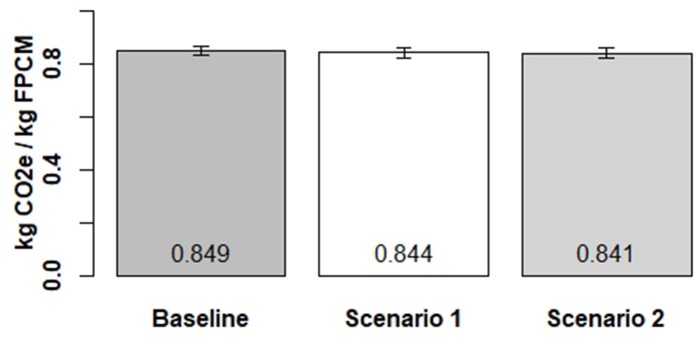
Effect of milk loss reduction on GHG emissions in Scenario 1 and Scenario 2, mean and standard error.

Carbon footprints were carried out to determine the effect on GHG emissions on farm if milk loss could be reduced. Base footprints for milking cows on the surveyed farms averaged 0.849 kg FPCM/kg CO_2_e (Figure 2). In Scenario 1, disease prevention, leading to a 20% reduction in total withdrawal days meant net emissions slightly increased because of the need to cool increased quantities of milk. However, emissions per unit decreased to an average of 0.844 kg in Scenario 1 (Figure 2). Reducing withdrawal days by 20% equated to lowering the footprints by 0.6% on average.

In Scenario 2 additional milk sales brought about by fewer infrastructure and bulk tank losses reduced the farm footprints to an average to 0.823 kg (Figure 2), a reduction from the baseline of 1%. In Scenario 1, milk sales from farms increased by an average of 1,963 liters, which equated to additional income of £574. In Scenario 2 milk sales increased by 5,330 liters which equated to £1,559 of additional income.

On average, farmers who housed all year round managed larger herd sizes and attained higher yields than those managing composite or part summer housing (Table 4). Withdrawal periods for mastitis in AYR housed herds were, on average, 1 day longer and quantities withdrawn greater than in composite or part summer systems. When compared with composite and part summer housed systems, farmers operating AYR housed management regimes sold a greater share of milk because this system type had proportionally fewer losses of milk stemming from infrastructure, and fewer rejections by processors (Table 5).

**Table 4:** Farm indicators by management system type (mean).

	**Farms (*n*)**	**Cows (*n*)**	**Yield /cow (l)**	**CM withdrawal (days)**	**Rejected/cow (l)**
Composite	21	158	7,764	5.3	33
AYR Housed	12	289	10,026	6.4	82
Part Summer	10	205	7,420	5.3	29

*CM, Clinical mastitis*.

**Table 5:** Descriptive statistics of proportional losses by management system type.

	**Composite**	**Part summer**	**House AYR**
Sold	0.980	0.984	0.986
Retained	0.0092	0.0036	0.0039
Infrastructure losses	0.0051	0.0065	0.0018
Rejected by farmer	0.0048	0.0037	0.0080
Bulk tank losses	0.0002	0.0012	0.0002
Rejected by processor	0.0008	0.0013	0.0004

*AYR, All Year Round*.

## Discussion

This research evaluates causes and amounts of milk loss in primary production on Scottish dairy farms by gathering comprehensive data and conducting a partial LCA. A small proportion of milk is not sold for a variety of reasons, which are ultimately governed by management practices and farm infrastructure. Results captured flows of milk on dairy farms and showed infrastructure and rejected milk influencing losses, which can stem from disease events through to bulk tank failures. Opportunities for improvement were modeled by assessing possible GHG emission reductions corresponding to a 20% decrease in antibiotic use and also by reducing losses further from farm infrastructure. A 20% decrease in antibiotic use is consistent with current targets for reductions in total usage in the dairy sector (25).

Survey results were fairly representative, our sample averages indicated a herd size of 205 and an average annual yield per cow of 8,315 liters, which were close to Scottish averages in 2016 of 199 cows and 7,053 liters (42). Average milk constituents, calving interval, replacement rate and SCC, shown in Table 3, are comparable with average key production indicator (KPI) results reported for UK Holstein herds of 4.02% fat, 3.28% protein, 27% replacement, a 400 day calving interval and a 178 herd SCC (43). Whilst our sample represented only 5% of total farms, the similarity of survey response averages and industry averages suggests that the data gathered is not atypical.

Improving the efficiency of food production can be driven by associations between agricultural management systems and global issues, such as climate change, soil erosion, air pollution, and a loss of biodiversity (44, 45). Carrying out an LCA for agricultural systems may add a layer of complexity because farms utilize multiple inputs, such as natural resources and land and can adopt regionally diverse management practices (46). LCA's of milk production focusing on environmental attributes have been carried out to assess different types of dairy management, such as grazing and housed systems, or to compare organic and conventional regimes (47–50). LCA's can focus on system attributes such as the environmental impact of mastitis (17) or extend the boundary beyond the farm gate to include processing and retail (48, 51). Even though milk loss is accounted for in some studies it is not the focus. As far as we are aware, other figures detailing multiple sources and quantities of milk not sold from dairy farms, are not available.

Where broad data are available, the cause of rejected milk is reported as being mainly due to mastitis, because this disease is the most predominant on dairy farms leading to milk withdrawal (1). Survey results show that milk rejections stemming from treatments for health events such as lameness, uterine and other disorders led to almost a quarter of all withdrawal days on surveyed farms. Red Tractor, a UK food assurance scheme has reformed its standards on the use of antibiotics on dairy farms. Since 2018, Red Tractor requires accurate recording and staff training in the use of medicines, with an annual review of use to qualify for membership (52). Since this survey was carried out, usage of the highest priority, critically important antibiotics on UK dairy farms should now be directed by a veterinary practitioner and must be used as a last resort. One consequence of the restrictions on antibiotic use could be that zero withdrawal critically important antibiotics are substituted with treatments that do require milk rejection. This possible increase in withdrawal days suggest that health events such as metritis and lameness should be included to accurately estimate quantities of milk loss on farm in the future.

In the US a third of dairy farms are thought to feed waste milk to calves, and one UK survey of waste milk feeding practices reported a figure of 83% (16, 53). Our results showed 19% of farmers feeding rejected milk to calves in Scotland. This could be due to awareness of antimicrobial resistant bacteria in feces (20, 21) and pressure from milk buyers to ban the practice. It is possible, however, that pressures could lead to underreporting. Livestock manure applied to soil as a fertilizer provides organic matter, nutrients and microbial populations to grasslands, and is a source of GHG's as organisms decompose and recycle the feces (54, 55). Micro biological and chemical changes to livestock manure as a result of antibiotic treatments have been shown to alter the microbiota of dung beetles, and lead to increased manure methane emissions (56). Further effort should be made to quantify the undesirable consequences of spreading slurry containing waste milk as there could be additional and unforeseen ecological effects.

Further along the FSC, UK pasteurized milk waste stemming from households, shops, transit and processing was estimated to be 7% of all liquid retail sales. Our results indicated 0.55% of milk production was rejected due to antibiotic residue across 5% of the farms surveyed, which averaged over 10,000 liters per farm. If bulk tank rejection and loss amounts were included this would raise the figure further. Information regarding rejection of deliveries at processor gate would improve overall estimates. Greater volumes of milk were found to be rejected in AYR housed systems which had larger herd sizes, although proportionally these farms sold more than composite systems because the amounts of milk intentionally retained and lost from infrastructure were lower. Some farmers reported zero milk losses from infrastructure because washings were used to feed calves. Whilst this practice saves waste, some may argue that the quality and constituents of the feed may be unpredictable.

Product GHG emissions were shown to be reduced by 0.6 and 3.1%, respectively, if losses from rejected milk and from farm infrastructure were decreased. Reducing withdrawal days through prevention and management of disease, spillages, and accidents, could be achieved on farm through a combination of precision technologies and improved management procedures. For example, treatments for mastitis may be unnecessary if an early diagnosis of the disease brought about through technological and management solutions could be achieved. A reduction in losses from infrastructure could be achieved by implementing strict management routines along with up to date technology such as alarms and modern filters in the parlor system. Methods to reduce milk loss in primary production could be targeted by dairy management system type.

Regardless of system type, dairy cows are dried off approximately 8 weeks prior to calving. In the UK until recently, all cows were given routine prophylaxis in the form of antibiotic dry cow treatment and teat sealant. However, widespread use is now being discouraged and non-antibiotic teat sealants are available. If a cow receives prophylaxis at dry off, after calving the colostrum can contain residue for 3 days and Brunton et al. (16) reported that 96% of farms used dry cow antibiotic tubes with 85% of these applying to all cows. Scottish survey results show 98% of respondents utilize dry cow treatment, however only 49% treated all cows, as 49% of farmers used dry cow treatment selectively. Teat sealant was applied to all cows on 63% of farms, whereas it was used selectively on 16% of farms, whilst 21% of farmers did not use teat sealant. A third of respondents used dry cow treatment on all cows, and fed colostrum to calves directly from the cow.

## Conclusions

Survey results demonstrated that milk loss on dairy farms in Scotland stemmed from a range of health disorders and occurred at any stage from parlor to processor due to infrastructure, accidents and spills. Milk loss in primary production was not insignificant and measures should be taken to reduce losses, especially from disease and infrastructure. Progress made to reduce milk loss in primary production will also lower product GHG emissions and should improve food security. Ecological and animal health consequences of milk containing residues from veterinary treatment being fed to calves or entering slurry systems should be further researched.

## Data Availability Statement

The datasets generated from survey responses cannot be made available for confidentiality reasons.

## Ethics Statement

The scoping study accessed animal production data and Social Research Approval was obtained from Scottish Government.

## Author Contributions

MM and MH managed the survey delivery. MM carried out the survey analysis. LT, BT, MM, and MH contributed to concept, methodology, and manuscript preparation.

### Conflict of Interest

The authors declare that the research was conducted in the absence of any commercial or financial relationships that could be construed as a potential conflict of interest.
